# Evaluation of multiplex ARMS-PCR for detection of *Helicobacter pylori* mutations conferring resistance to clarithromycin and levofloxacin

**DOI:** 10.1186/s13099-020-00373-6

**Published:** 2020-07-10

**Authors:** Yangxia Li, Tangshan Lv, Chaochao He, Hongyan Wang, David S. Cram, Linfu Zhou, Jun Zhang, Weiqin Jiang

**Affiliations:** 1 Key Laboratory of Clinical In Vitro Diagnostic Techniques of Zhejiang Province, Department of the First Affiliated Hospital, School of Medicine, Zhejiang University, Hangzhou, 310003 China; 2 Zhejiang Molebioscience Ltd, Hangzhou, 310058 China; 3 Department of Biochemistry and Molecular Biology, Zhejiang University School of Medicine, Hangzhou, 310058 China; 4 Department of Gastroenterology, Huzhou Central Hospital & Affiliated Central Hospital, Huzhou, 313000 China; 5 Department of Medical Oncology, First Affiliated Hospital, College of Medicine, Zhejiang University, Hangzhou, 310003 China

**Keywords:** *Helicobacter pylori*, Real-time ARMS-PCR, Clarithromycin resistance, Levofloxacin resistance

## Abstract

**Background:**

*Helicobacter pylori* bacterium is a major cause of gastritis. With increasing use of antibiotics to treat infections, mutation resistant strains have emerged in most human populations. To effectively treat patients to help resolve infections, the clinician needs information on the antibiotic susceptibility profile of the infection. Therefore, a rapid and accurate test is required to provide this information. To address this issue, we designed and validated a real time multiplex ARMS-PCR assay for rapid detection of highly prevalent *H. pylori* clarithromycin and levofloxacin resistance mutations. The aim of the study was to evaluate the analytical and diagnostic sensitivity and specificity of ARMS-PCR, using direct Sanger sequencing of the known resistance mutations as the gold standard.

**Results:**

In preliminary studies using a defined number of plasmids with clarithromycin and levofloxacin resistance mutations, the analytical sensitivity of our ARMS-PCR assay was 50 plasmid copies, equating to around 50 bacterium in a gastric biopsy sample. In terms of specificity, the assay was highly specific for the targeted resistance mutations. The assay was also able to reliably and efficiently detect heteroresistance of clarithromycin and levofloxacin mutations, even at a disproportional ratio of 1:1000. From the analysis of 192 samples with suspected *H. pylori* infections, the diagnostic sensitivity and specificity of the assay was very high for detection of clarithromycin resistance (100% and 100%), levofloxacin resistance (98.04% and 95.04%) and clarithromycin and levofloxacin double resistance (100% and 96.91%). Amongst the 74 patients diagnosed antibiotic resistance bacteria, 23 (31.1%) had clarithromycin resistance, 21 (28.4%) had levofloxacin resistance and 30 (40.5%) had double resistance. From sample receipt to results, our single tube assay could be routinely completed in under 2 h.

**Conclusions:**

Our assay demonstrated high diagnostic sensitivity and specificity for detection of clarithromycin and levofloxacin resistant *H. pylori*. Based on proven accuracy, together with high efficiency, scalability and low cost, our assay has useful clinical utility for rapid diagnosis of clarithromycin and levofloxacin resistant *H. pylori* infections. Our assay results will provide patients with a clear diagnosis, enabling the treating clinician to administer the most effective antibiotic regimen to help the clear the infection.

## Background

*Helicobacter pylori* (*H. pylori*) has colonized the upper gastrointestinal tract of over 50% of the world’s population [1]. While the vast majority of infected people remain asymptomatic, the bacterium can cause gastritis and peptic ulcers. In addition, *H. pylori* infections have been associated with a higher risk for development of gastric cancer. As such, the pathogen has been classified as a Group 1 carcinogen by the International Agency for Research on Cancer [2].

Simple treatment of *H. pylori* infections with antibiotics is the best strategy to alleviate ulceration and gastritis [3]. To achieve effective healing after an initial infection, it is particularly important to select an antibiotic that is bactericidal [4]. However in recent years, antibiotic resistance has increased alarmingly in strains of *H. pylori*, leading to a decline in the effectiveness of antibiotic treatment prescribed by clinicians. Consequently, before selection and administration of any antibiotic treatment it has been recommended by the III Working Group for the management of Helicobacter pylori infection [5] and the American College of Gastroenterology (ACG) [6] that a molecular test on a gastric biopsy sample should be performed for determining antibiotic susceptibility.

For many years, the antibiotic clarithromycin was usually used as a first-line regimen for treatment of *H. pylori* infections. When clarithromycin proved largely ineffective, a bismuth quadruple therapy or levofloxacin triple therapy was chosen as the second-line treatment option [3]. Subsequently, a significant proportion of patients were found to have new infections caused by clarithromycin and levofloxacin resistant *H. pylori*. Today in China alone, the resistance levels for clarithromycin and levofloxacin have reached 20%–50%, and thus new antibiotics are needed to treat *H. pylori* infections [7]. Molecular studies have shown that resistance to clarithromycin is largely due to three point mutations within the peptidyltransferase-encoding region of the 23S *rRNA* gene, namely: 2142A > C, 2142A > G and 2143A > G [8]. On the other hand, resistance to levofloxacin is mainly associated with *gyrA* gene variants 259A > T and 261T/C > G/A at codon position N87 and variants 271G > A, 271G > T and 272A > G at codon position D91 [9].

Efficient and timely confirmation of *H. pylori* infection and concomitant analysis of antibiotic susceptibility is an important step to prescribe the most effective therapy regimen for patients. Currently, although this can be simply performed by bacterial culture using the antibiotic susceptibility E test, the test lacks sensitivity and takes around 1 week for informative results [10]. Several alternative molecular approaches have been developed including PCR and reverse dot blot hybridization [11–13], real-time PCR methods with melt curve analysis [8] and more recently an ARMS-PCR based method [14]. However, while these tests exhibited a high degree of sensitivity and specificity, there efficiency is not sufficient to provide timely laboratory results and the designs only focused on detecting common mutations for either clarithromycin or levofloxacin resistant *H. pylori*.

To improve efficiency and coverage, we developed a novel real-time multiplex ARMS-PCR approach that has the capacity to directly detect the presence of *H. pylori* as well as clinically significant clarithromycin and levofloxacin resistance mutations in a single tube assay, providing rapid and accurate results within 2 h. In a pilot study of 192 gastric biopsies from patients suspected to have *H. pylori* infections, we compare the diagnostic performance of our new assay for detection of clarithromycin and levofloxacin against Sanger sequencing that directly reveals the precise antibiotic resistance mutations.

## Methods

### Patients

Between September and December 2018, a total of 192 patients presented at the Gastroenterology clinic of the Huzhou Central Hospital with symptoms of gastritis. There were 95 female and 97 male patients, with an average age of 46.7 years (range 34–69 years). Following gastroscopy, biopsy samples were taken from the gastric antrum. By H&E staining, all samples showed gastric inflammation. Apart from inflammation, none of the samples showed histological evidence of gastric cancer. All patients provided written informed consent for gastroscopy and testing biopsy samples for antibiotic resistant *H. pylori*. The study was approved by the Ethics Committee for clinical drug trials, Huzhou Central Hospital (Approval number 2018-006-02).

### Preparation of genomic DNA from gastric biopsy samples

DNA from gastric biopsies was extracted using a nuclear acid extraction kit (Jiangsu Mole Bioscience, Taizhou). Briefly, a gastric biopsy sample (~ 10 mg) was placed in an eppendorf tube with 100 μL lysis buffer, vortexed and then held at 99℃ for 10 min. After cooling to room temperature the tube was centrifuged for 10 min. The DNA in 5μL of supernatant (~ 5% of the sample) was used directly as the input template for molecular analysis.

### Multiplex ARMS-PCR real-time assay

**Fig. 1:**
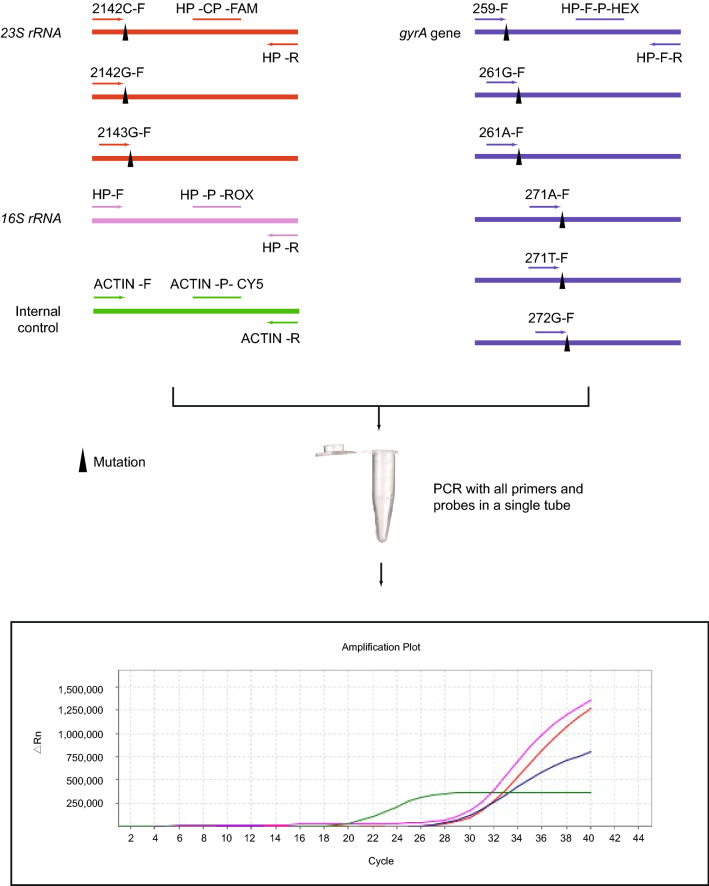
Multiplex ARMS-PCR assay for detection of *H. pylori* clarithromycin and levofloxacin resistance

The key steps of a novel one tube multiplex ARMS-PCR real-time assay that was designed and optimized for detection of *H. pylori* and clinically significant antibiotic resistance mutations are shown in Fig. 1. Relevant ARMS-PCR primers for amplification of specific regions of the *16S rRNA* gene (diagnostic for *H. pylori*) and the *23S rRNA* and *gyrA* genes (diagnostic for clarithromycin and levofloxacin resistance) are listed in Table 1. All primers and labelled probes were synthesized by Thermofisher (USA). PCR assays were performed in a single tube on the ABI7500 machine (ABI, USA) using 5 μL of sample lysate, with an initial cycle of 42 °C for 2 min, then one cycle of 95 °C for 2 min, followed by 40 cycles of 95 °C for 10 s and 58 °C for 45 s. The ARMS-PCR-specific products were detected at the annealing/amplification phase of the PCR reaction using fluorescent Taqman gene probes for *16S rRNA* (ROX), *23S rRNA* (6-FAM), *gyrA* (HEX) and, *β*-*actin* (CY5) used as the positive control gene.

**Table 1:** Primers and probes for ARMS-PCR and Sanger sequencing

Methods	Gene	Primers/probes	Sequence (5′-3′)	Modification
ARMS-PCR	*16S rRNA*	HP-F	CCCATCAGGAAACATCGCTTCA	
		HP-R	TCCACTATGCTGGAGAATTGGCTA	
		HP-P-ROX	TGCTTGCCACGCCATCCATCACATCA	5′ROX, 3′BHQ1
	*gyrA*	259-F	CACCCCCATGGCGGTT	
		261G-F	CCCCATGGCGATACG	
		261A-F	CCCCATGGCGATACA	
		271A-F	CATGGCGATAATGCGGTTTGTA	
		271T-F	CATGGCGATAATGCGGTTTGTT	
		272G-F	ACCCCCATGGCGATAATGCGGTTTATCG	
		HP-F-R	ACTCGCCTTAGTCATTC	
		HP-F-P-HEX	CGTTATCGCCATCAATAGAGCCAA	5′HEX, 3′BHQ2
	*23S rRNA*	2142C-F	TCCTACCCGCGGCAAGACTGC	
		2142G-F	TTCCTCCTACCCGCGGCAAGACAGG	
		2143G-F	CCTACCCGCGGCAAGACGACG	
		HP-C-R	GGATGACTCCATAAGAGCCAAAGC	
		HP-C-P-FAM	CGTCTTGCCGCGGGTAGGAGGAATTTTCAC	5′FAM, 3′BHQ1
	Internal control	ACTIN-F	CCCATCTACGAGGGCTACGC	
		ACTIN-R	GGATCTTCATGAGGTAGTCGGTCAG	
		ACTIN-P-CY5	CCATCCTGCGTCTGGACCTGGCTGGC	5′CY5, 3′BHQ2
Sanger sequencing	*23S rRNA*	23S-F	TCCCTCCCGACTGTTTACCAAA	
		23S-R	GCCATTACACTCAACTTGCGATTTC	
	*gyrA*	gyrA-F	GCAAGATAATTCAGTCAATGAAACA	
		gyrA-R	CCACAGCGATCCCATTAGC	

Analytical sensitivity of our assay was assessed using positive and negative control plasmid samples. Plasmid pGH clones containing the wildtype *23S rRNA* gene and mutated variants 2142C, 2142G, 2143G and, plasmid clones containing the wild *gyrA* gene and variants 259T, 261A, 261G, 271A, 271T and 272G were supplied by the Shanghai Generay Biotech. The wildtype sequences of the *16S rRNA*, *23S rRNA* and *gyrA* genes were taken from the reference *H. pylori* strain 26695. Plasmid preparations were serially diluted to different DNA concentrations, and then added to lysis buffer containing DNA from an extract of *H. pylori* negative gastric mucosa to mimic clinical sample conditions. Twenty replicates of 5 μL aliquots containing 5, 50 and 500 copies of each plasmid was analyzed in real-time ARMS-PCR assays to calculate analytical sensitivity. For testing levofloxacin and clarithromycin heteroresistance, samples were prepared with pGH plasmids containing a mutated *23S rRNA* gene and a mutated *gyrA* gene in different ratios (1:0.1, 1:0.01 and 1:0.001) and, for analysis, mixed with DNA extracted from *H. pylori* negative gastric biopsies.

### Sanger sequencing of *23S rRNA* and *gyrA* genes

The PCR primers used to generate *23S rRNA* (556 bp) and *gyrA* (552 bp) gene amplicons for sequencing are shown in Table 1. Standard PCR conditions using 5 μL of sample lysate as the starting DNA template were one cycle of 42 °C for 2 min, one cycle of 95 °C for 2 min, 45 cycles of 95 °C for 10 s, 58 °C for 45 s and then a final cycle of 72 °C for 35 s. Amplicons were gel purified and Sanger sequenced using 23S-R and gyrA-R primers (Table 1). Sequences were aligned to the *H. pylori* reference sequence (strain 26695) and analyzed for antibiotic resistance mutations using Chromas software (Technelysium, version 2.22).

### Statistical analysis

The sensitivity, specificity, positive predictive value of our ARMS-PCR assay was compared against gold standard Sanger Sequencing results.

Sensitivity = True positives/(True positives + False negatives) = ${\text{a/a + c}}$

Specificity = True negatives/(True Negatives + False Positives) = ${\text{d/b + d}}$

Positive predictive value = True positives/Total positives = ${\text{a/a + b}}$

A kappa test was also used to determine concordance between ARMS-PCR real-time PCR results and Sanger sequencing results.

$${\text{Kappa value}}\, = \,\left( {{\text{P}}_{\text{o}} - {\text{P}}_{\text{e}} } \right) /\left( {1 - {\text{P}}_{e} } \right)$$

$${\text{P}}_{\text{o}} = \left( {{\text{a}} + {\text{d}}} \right)/\left( {\text{a + b + c + d}} \right)$$

$${\text{P}}_{\text{e}} = \left[ {\left( {\text{a + c}} \right)\left( {\text{a + b}} \right){ + }\left( {\text{b + d}} \right)\left( {\text{c + d}} \right)} \right] /\left( {\text{a + b + c + d}} \right)^{2}$$

where a = true positives, b = false positives, c = false negatives and d = true negatives.

## Results

### Optimization of multiplex ARMS-PCR test

**Fig. 2:**
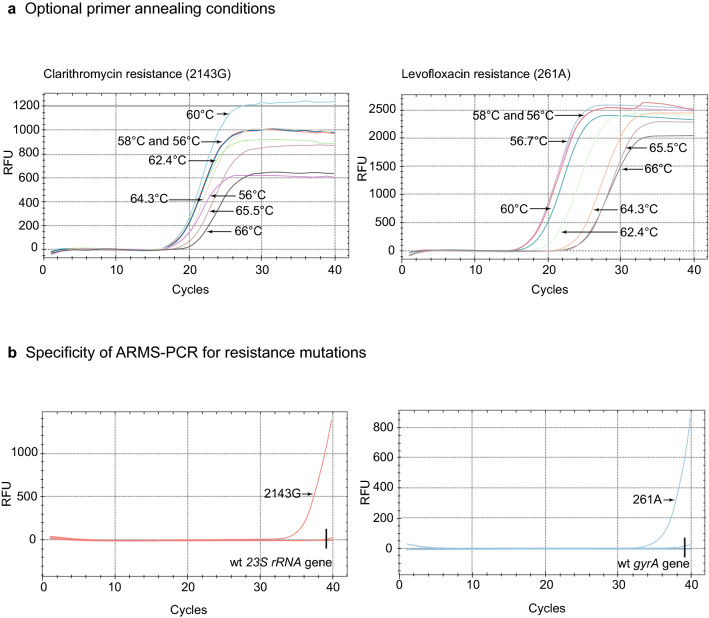
Optimization of key parameters of multiplex ARMS PCR assay. **a** Optimal primer annealing conditions. Mutations 2143G (clarithromycin resistance) and 261A (levofloxacin resistance) were used in the test as a starting input of 10^8^ plasmids for ARMS-PCR. An annealing temperature of 58 °C provided optimal amplification of the mutation sequences. **b** Specificity of ARMS-PCR for resistance mutations. Wildtype *23S rRNA* and *gyrA* sequences were tested at a starting input of 10^8^ plasmids. Positive control mutations 2143G and 261A mutations were tested at a starting input of 500 plasmids. The ARMS-PCR test was highly specific, only detecting the mutation sequences

A detailed series of experiments were performed to optimize the sensitivity and specificity of the multiplex ARMS-PCR test for detection of clarithromycin and levofloxacin resistance mutations. The key factors examined were the PCR reaction conditions and the best annealing temperature for the ARMS-PCR primers (Fig. 2a, example *23S rRNA* mutation 2143G and *gyrA* mutation 261A). Based on the minimum number of cycles to initiate productive PCR and high yield, 58 °C proved to be the optimal annealing temperature for detection of all clarithromycin and levofloxacin mutations covered by the test. In further experiments, we showed that using an input of up to wildtype 10^8^ plasmids, that the multiplex ARMS-PCR test did not non-specifically amplify the wildtype *23S rRNA* and *gyrA* wildtype sequences in the presence of positive control plasmids containing the *23S rRNA* 2143G and *gyrA* 261A mutations (Fig. 2b). These experiments indicated that our multiplex ARMS-PCR test was highly specific for detection of clarithromycin and levofloxacin resistance mutations.

### Analytical sensitivity of the ARMS-PCR real-time assay

**Fig. 3:**
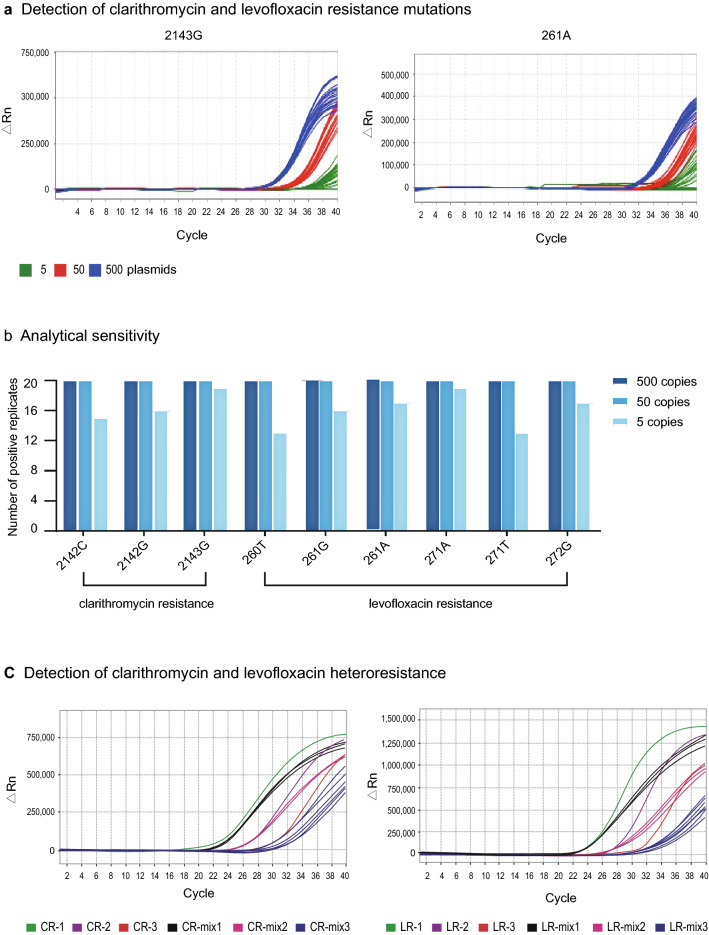
Analytical sensitivity of the multiplex ARMS-PCR assay.**a** Detection of clarithromycin resistance mutations. **b** Analytical sensitivity. For all the clarithromycin and levofloxacin mutations tested, the minimum detection sensitivity was 50 copies. **c** Detection of clarithromycin and levofloxacin heteroresistance. For modelling heteroresistance, ARMS-PCR reactions were performed with clarithromycin resistance mutation 2143G and levofloxacin resistance mutation 261A, respectively. LR-1, LR-2 and LR-3 denotes 261A with a starting input of 10^6^, 10^5^ and 10^4^ plasmids, respectively. CR-1, CR-2 and CR-3 denotes 2143G with a starting input of 10^6^, 10^5^ and 10^4^ plasmids, respectively. LR-mix1, LR-mix2 and LR-mix3 indicate 261A:2143G ratios of 1:0.1, 1:0.01 and 1:0.001, respectively. CR-mix1, CR-mix2 and CR-mix3 indicate 2143G:216A ratios of 1:0.1, 1:0.01 and 1:0.001, respectively. At all mutation ratios, both mutation sequences were efficiently amplified, indicating high sensitivity of the test for detecting heteroresistance

Multiple replicates (n = 20) of plasmids containing the clarithromycin and levofloxacin antibiotic resistance mutations were analyzed by the real-time ARMS-PCR assay (Fig. 3a, example clarithromycin and levofloxacin resistance mutations 261A and 2143G). Overall, detection of the nine antibiotic resistance mutations at 5 copies per reaction was inconsistent (Fig. 3b). At higher mutation levels of 50 and 500 copies per reaction, there was consistent detection of all antibiotic resistance mutations in each of the 20 replicates. Taken together, we defined the minimum analytical sensitivity of the assay as 50 mutation copies for detection of clarithromycin and levofloxacin resistance.

For testing clinical situations where there is heteroresistance (both clarithromycin and levofloxacin resistant *H. pylori*), samples were prepared with different ratios (1:0.1, 1:0.01 and 1:0.001) of plasmids containing a mutated *23S rRNA* and *gyrA* gene sequence. For these experiments, the ratio value of 1 was set at a starting input of 10^7^ plasmids. From the experimental results (Fig. 3b, example clarithromycin mutation 2143G and levofloxacin mutation 261A), even at a disproportionate ratio of 1:1000, the 2143G and 261A sequences were efficiently amplified, even though there was some delay in amplification of the lowest level mutation. Similar PCR amplification trends were observed for other mutation combinations modelled for heteroresistance. On this basis, we concluded that our multiplex test was capable of reliably detecting the presence of both clarithromycin and levofloxacin resistant *H. pylori* in clinical samples.

### Clinical performance of multiplex ARMS-PCR assay

We analyzed 192 gastric biopsies from patients with suspected *H. pylori* infections, comparing results to Sanger sequencing as the gold standard (Table 2). The diagnostic sensitivity and specificity of the multiplex ARMS-PCR test for detection of *H. pylori* bacterium was 100% and 95%, respectively, with 4 false positives and no false negatives. In regard to the diagnosis of antibiotic resistance, the assay had a sensitivity of 100% (no false negatives) and a specificity of 100% (no false positives) for detection of clarithromycin resistance, a sensitivity of 98% (1 false negative) and a specificity of 95% (7 false positives) for detection of levofloxacin resistance and a sensitivity of 100% (no false negatives) and a specificity of 97% (5 false positives) for double resistance. In all assay comparisons, the kappa values ranged from 0.9 to 1.0, indicating a high degree of concordance of ARMS-PCR and Sanger sequencing assay results for detection of *H. pylori* and antibiotic resistance.

**Table 2:** Performance of multiplex ARMS-PCR for detection of *H. pylori* infection and antibiotic resistance in 192 gastric biopsy samples

Parameters	*H. pylori*			Clarithromycin resistance			Levofloxacin resistance			Clarithromycin and Levofloxacin resistance		
	Sequencing			Sequencing			Sequencing			Sequencing		
	+	-		+	-		+	-		+	-	
+	108	4	112	53	0	53	50	7	57	30	5	35
_	0	80	80	0	139	139	1	134	135	0	157	157
	108	84	192	53	139	192	51	141	192	30	162	192
Sensitivity	100%			100%			98.04%			100%		
Specificity	95.24%			100%			95.04%			96.91%		
PPV	97.92%			100%			95.83%			97.40%		
Kappa	0.96			1.00			0.90			0.91		

### Precision of the ARMS-PCR assay for detection of antibiotic resistance

**Fig. 4:**
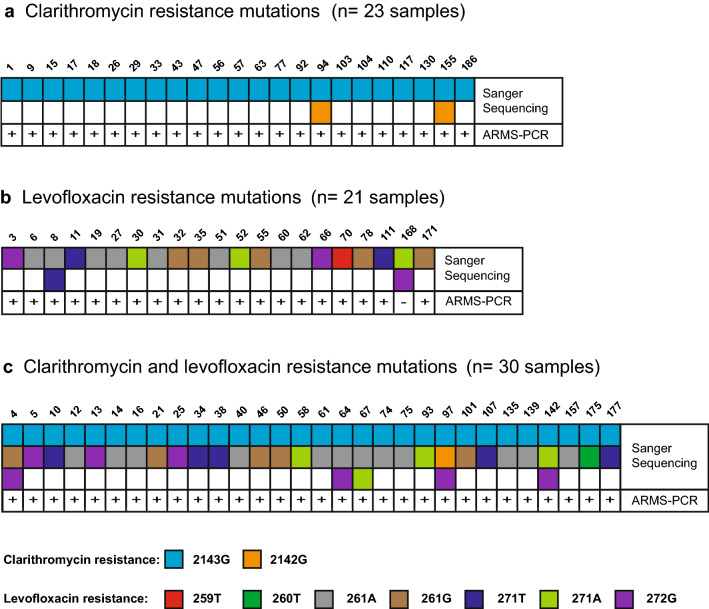
Clarithromycin and levofloxacin resistance mutations detected in the 74 positive samples. Sanger sequencing revealed the precise mutations associated with antibiotic resistance. There was a strong positive correlation of specific *23S rRNA* and *gyrA* mutations with a positive ARMS-PCR result for clarithromycin and levofloxacin resistance

The sensitivity and specificity of the multiplex PCR assay for the 74 antibiotic resistant samples was also calculated to determine the precision of the test (Table 3). Detection sensitivities were high for clarithromycin (100%), levofloxacin (98%) and clarithromycin/levofloxacin (100%) resistance. Based on the Sanger sequencing results for the antibiotic resistance mutation combinations in the 74 samples (Fig. 4), 23 samples had clarithromycin mutations, 21 had levofloxacin mutations and 30 had both clarithromycin and levofloxacin mutations. Of the 56 *23S rRNA* mutations identified, 2143G was common (53, 94.6%) and 2142G was rarer (3, 5.4%). In the case of the 56 *gyrA* mutations identified, the most common was 261A (20, 35.7%), followed by 261G (10, 17.9%), 272G (9, 16.1%), 271T (8, 14.2%), 271A (7, 12.5%), 259T (1, 1.8%) and 260T (1, 1.8%). Although ARMS-PCR was only designed to detect positive samples with clarithromycin and levofloxacin resistant *H. pylori*, based on direct sample comparison to Sanger sequencing mutations, the results suggested that multiplex ARMS-PCR was highly precise for detection of the antibiotic resistance mutations covered in the scope of the molecular test design. Despite the inferred high precision, the specificity of the test amongst the 74 antibiotic resistance samples was variable (Table 3). While the specificity for detection of clarithromycin resistance was 100%, specificities were significantly lower for detection of levofloxacin resistance (69.6%) and clarithromycin and levofloxacin resistance (88.6%) resistance.

**Table 3:** :Performance of multiplex ARMS-PCR for detection of antibiotic resistance in the 74 positive samples

Parameters	Clarithromycin resistance			Levofloxacin resistance			Clarithromycin and Levofloxacin resistance		
	Sequencing			Sequencing			Sequencing		
	+	–		+	–		+	–	
ARMS-PCR									
+	53	0	53	50	7	57	30	5	35
_	0	21	21	1	16	17	0	39	39
	53	21	74	51	23	74	30	45	74
Sensitivity	100%			98.04%			100%		
Specificity	100%			69.57%			88.64%		
PPV	100%			89.19%			93.24%		
Kappa	1.00			0.73			0.86

### Spectrum of *H. pylori* antibiotic resistance

**Fig. 5:**
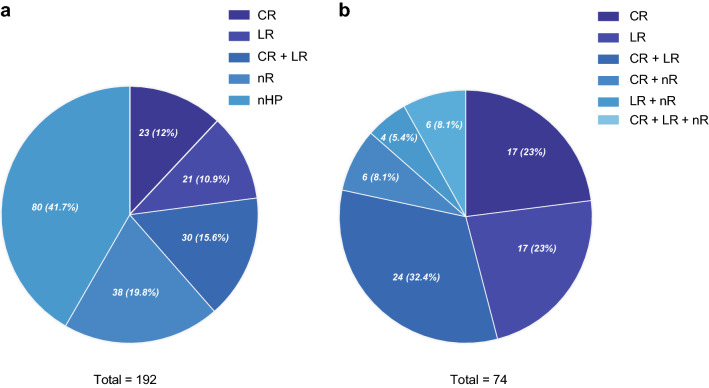
*H. pylori* antibiotic resistance spectrum. **a** Distribution of antibiotic resistance in 192 samples with suspected *H. pylori.***b** Distribution of antibiotic resistance in 74 antibiotic resistant samples. CR, clarithromycin; LR, levofloxacin; CR + LR, clarithromycin and levofloxacin; nR, no resistance; nHP, no *H. pylori* detected

From the global analysis of the 192 samples with suspected *H. pylori* infections, there were 80 (41.7%) samples with non-detectable *H. pylori*, 23 (9.4%) samples classified with clarithromycin resistance, 21 (10.9%) with levofloxacin resistance, 30 (18.2%) with double resistance and 38 (19.8%) with no resistance (Fig. 5a). Sanger sequencing revealed finer details of the 74 positive antibiotic resistant patient samples, with the majority (58, 78.4%) having only antibiotic resistant *H. pylori* infections and a minority (16, 21.6%) having mixed infections of antibiotic resistant and antibiotic non-resistant *H. pylori* (Fig. 5b).

## Discussion

In this current study, we introduce a multiplex ARMS-PCR assay for detection of clarithromycin and levofloxacin resistant *H. pylori* infections from gastric biopsy samples. In preliminary validation studies using plasmids with the known antibiotic resistance mutations, we showed that the analytical sensitivity of the test was at least 50 DNA copies. In addition, in samples with mixtures of mutation and wildtype sequences, we showed that the test was highly specific for detection of the mutation sequences. Further, by creating artificial plasmid mixtures mimicking clinical samples, the test was able to reliably and efficiently detect heteroresistance at ratios as extreme as 1:1000. In a study of 192 gastric biopsy samples, compared to gold standard Sanger sequencing assay which definitively identifies antibiotic resistance mutation sequences, our ARMS-PCR assay exhibited high diagnostic sensitivity and specificity for detection of clarithromycin, levofloxacin and double resistant *H. pylori*.

The multiplex ARMS-PCR we built and optimized was designed for detection of *H. Pylori* infection as well as for determining whether the bacterium were resistant to clarithromycin and levofloxacin antibiotics. Of the 192 biopsy samples from patients with suspected *H. pylori* infections that we studied, all had histological evidence of *H. pylori* infection. However, by genetic testing the *16S rRNA* gene sequence by Sanger sequencing and ARMS-PCR, 112 of the 192 samples (58.3%) were confirmed with *H. pylori* infection. There are a number of potential factors that could explain a negative finding of *H. pylori* in the presence of gastritis, including gastritis due to other causes such as such as EBV infection [15] and autoimmune gastritis [16], natural resolution of the infection, a poor biopsy sample, and a limitation on the sensitivity of the genetic tests used for detecting *H. pylori*. For example, in this study, we showed that the minimum analytical sensitivity of multiplex ARMS-PCR was 50 bacterium. Thus, in some of the negative samples, it is possible that if there were any bacterium present, the numbers in actual biopsy sample taken may have been below the limit of detection of the genetic tests.

Apart from the high precision of the test established in validation and clinical studies, the multiplex ARMS-PCR assay we developed and validated has considerable additional advantages for the analysis of clinical samples for *H. pylori* antibiotic resistance. Firstly, from sample receipt to a clinical report, the assay takes around 2 h to perform. This allows the treating clinician to have same day answers, allowing prescription of effective antibiotic therapy for the patients. Second, excluding labour, the reagents to perform the assay for one sample costs less than 1 USD in China and, by using a 96 micro titer plate format, it will be possible to analyze at least 90 samples in single wells simultaneously with the appropriate positive and negative controls. Further, time and cost parameters are more favorably for our new assay compared to all other published methods [8, 10–14, 17]. Thus, our new assay would be more compatible with the current work flow in large hospitals that deal with treatment of patients with gastritis on a daily basis. In addition, since our assay is based on a multiplex PCR format, it has inherent capacity for expansion to detect other antibiotic resistant *H. pylori*, by incorporating ARMS-PCR primers for other clinically significant mutations with alternative fluorescent Taqman probes. The assay we designed and validated has specific application for diagnosis of patients with infections of antibiotic resistant *H. pylori*. One perceived deficiency in the design of our assay compared to using direct sequencing as an assay was the inability to detect mixed infections of non-antibiotic and antibiotic resistant *H. pylori*, which occurred in 21.6% of antibiotic resistance cases. However, for the clinician, just the knowledge of what antibiotic resistant *H. pylori* are present, is the critical information that our assay provides, so that patient treatment can be immediately prescribed with a bactericidal antibiotic that will effective against both the resistant and non-resistant *H. pylori*.

While our ARMS-PCR assay exhibited high performance for detection of clarithromycin and levofloxacin resistance, we identified several limitations for clinical application in a diagnostic pathology laboratory. Firstly, despite high sensitivity, the specificity of the test was significantly lower with approximately 5% of the resistance positive samples classified as false positives. False positives results can limit the choice of antibiotics selected for treatment and may in some cases lead to ineffective treatment of the infection. Thus, on occasions, some patients may need a second course of an alternative antibiotic to resolve the infection. Secondly, while detection of the major clarithromycin and levofloxacin resistance mutations are built into our test, the strain J99 with the alternative levofloxacin resistance mutation 261C at position N87I [18] is not detectable with our test. Thus, patients with the 261C *H. pylori* strain may be unknowingly administered levofloxacin by their clinician to treat the infection and similarly, will need to have a second treatment to resolve the infection with an alternative antibiotic. Thirdly, in the Asian population, there are occasional *H. pylori* infections with resistance mutations to other antibiotics such as amoxicillin and metronidazole [7], and thus are not detectable with our current test. Based on these limitations, it will be possible to expand the current test format to include new ARMS-PCR primers to additionally detect *H. pylori* infections associated with other rarer antibiotic resistant mutations present in the Asian population.

## Conclusions

We have designed and validated a simple one tube, rapid, accurate and low cost molecular assay suitable as a first tier diagnostic test for detection of clarithromycin and levofloxacin resistant *H. pylori* in gastric biopsy samples. This assay can provide same day results to the clinician so that a suitable and effective antibiotic can be immediately prescribed to help clear the infection and alleviate patient symptoms.

## Data Availability

All data generated or analyzed during this study are included in this published article.

## Abbreviations

*H. pylori*: *Helicobacter pylori*
ARMS-PCR: Amplification Refractory Mutation System PCR
